# Treatment of chronic anterior shoulder dislocation by open reduction and simultaneous Bankart lesion repair

**DOI:** 10.1186/1758-2555-2-15

**Published:** 2010-06-16

**Authors:** Alireza Rouhani, Amirmohammad Navali

**Affiliations:** 1 Orthopaedy department, Tabriz Medical & Sciences University, Tabriz, Iran

## Abstract

**Background:**

Untreated chronic shoulder dislocation eventually leads to functional disability and pain. Open reduction with different fixation methods have been introduced for most chronic shoulder dislocation. We hypothesized that open reduction and simultaneous Bankart lesion repair in chronic anterior shoulder dislocation obviates the need for joint fixation and leads to better results than previously reported methods.

**Methods:**

Eight patients with chronic anterior dislocation of shoulder underwent open reduction and capsulolabral complex repair after an average delay of 10 weeks from injury. Early motion was allowed the day after surgery in the safe position and the clinical and radiographic results were analyzed at an average follow-up of one year.

**Results:**

The average Rowe and Zarin's score was 86 points. Four out of eight shoulders were graded as excellent, three as good and one as fair (Rowe and Zarins system). All patients were able to perform their daily activities and they had either mild or no pain. Anterior active forward flexion loss averaged 18 degrees, external active rotation loss averaged 17.5 degrees and internal active rotation loss averaged 3 vertebral body levels. Mild degenerative joint changes were noted in one patient.

**Conclusion:**

The results show that the overall prognosis for this method of operation is more favorable than the previously reported methods and we recommend concomitant open reduction and capsulolabral complex repair for the treatment of old anterior shoulder dislocation.

**Level of Evidence:**

Therapeutic study, Level IV (case series [no, or historical, control group])

## Introduction

A glenohumeral joint that has remained dislocated for several days is called a chronic dislocation. These old dislocations most often are traumatic but frequently have been produced by a trivial injury as a result of the patient's increasing age and weakness and degeneration of the soft tissue about the shoulder joint such as the subscapularis and other rotator cuff tendons [1,2]. In younger patients unreduced dislocations often occur in those with alcoholism, seizures, or multiple trauma [3]. Usually the problems and complications of reduction are increased along with the chronicity of dislocation.

Open reduction and joint fixation has been suggested for most unreduced anterior dislocation of shoulder and different fixation methods have been used to prevent redislocation [4-6]. These fixation methods require long time immobilization and cause additional trauma to the articular surface of the humeral head and glenoid [4-6]. Little has been written on the results of these procedures.

Early shoulder motion improves cartilage nutrition and minimizes injury to the articular surfaces [7,8] but at the same time increases the risk of redislocation.

Given the documented success following Bankart lesion repair in recurrent anterior shoulder dislocation [9], we hypothesized that open reduction and simultaneous Bankart lesion repair of chronic anterior shoulder dislocation has the beneficial effect of safe early motion without the risk of redislocation.

## Materials and methods

Between November 2004 and September 2007, 15 patients with unilateral chronic anterior shoulder dislocation were referred to our clinic. Seven patients in whom closed reduction was possible were excluded from the study and the remaining eight patients were treated by open reduction and Bankart lesion repair. All patients were available for complete clinical and radiographic analysis at a minimum of 6 months postoperatively. Written informed consent was signed by all patients enrolled in the study. The patients included six men and two women with an average age of 42 years (range 17 to 75 years). The mechanism of injury was falling in all patients. Five dislocations involved the right arm and three the left arm. Five dislocations involved the dominant limb. The delay between dislocation and treatment ranged from 3 weeks to 5 months with an average of 10 weeks. Table 1 lists the demographic data of our patients.

**Table 1:** Demographic data's of patients

case	Age (year)	Duration of dislocation (weeks)	Duration of follow up (months)
1	25	6	18

2	23	8	6

3	36	17	15

4	65	3	12

5	35	7	12

6	17	22	6

7	75	8.5	11

8	61	7	18

**Figure 1:**
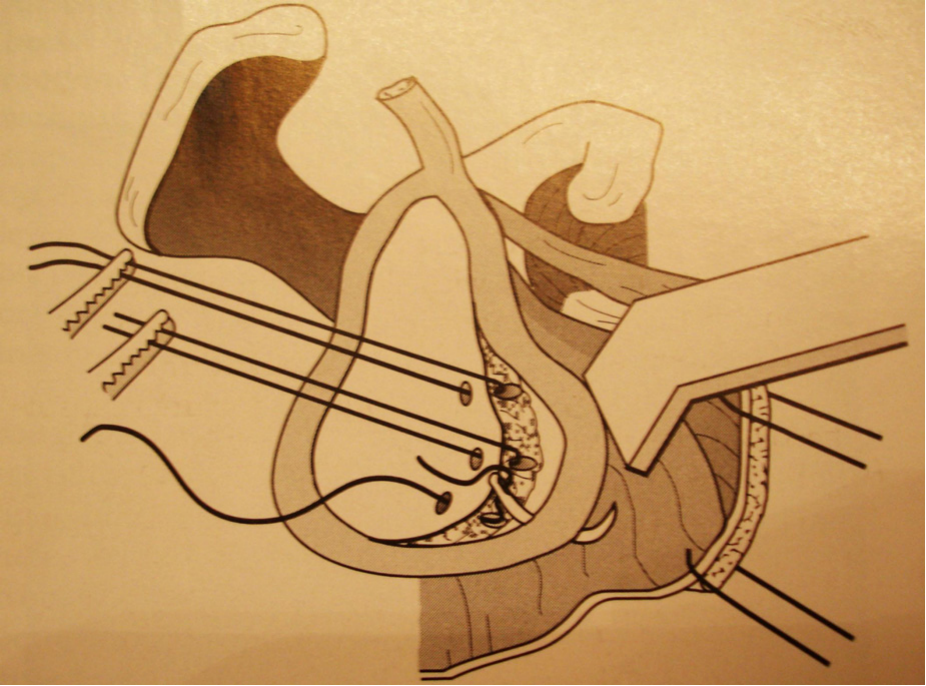
**Capsulolabral complex repair by transglenoid suture technique**.

Dislocations were diagnosed on anteroposterior radiographs and a definite diagnosis was made with an axillary projection. All dislocations had Hill-Sachs lesion with less than 40% of head involvement and all were nonengaging. Three cases had also greater tuberosity fracture. Surgery was performed with the patient in beach chair position. We used the anterior approach to the shoulder through the deltopectoral interval. Subscapularis tendon and capsule were cut in one layer and reduction was achieved with lateral traction and internal rotation. In two cases Coracoid osteotomy was done for better exposure. After reduction the capsulolabral complex was reinserted on to the anterior glenoid rim in all cases. Transglenoid suture with fiber wire no.2 was used for repair (figure 1). No bone graft was used in the anterior glenoid and humeral head and the capsule and Subscapularis tendon were repaired in internal rotation position. No joint fixation method was used following operative reduction. strong repair of capsule, subscapularis and Bankart lesion provided enough stability for postoperative rehabilitation. Greater tuberosity was fixed with transosseous suture in patients with greater tuberosity fracture. The upper limb was secured postoperatively according to Rowe and Zarins' sling method [10]. The arms were kept anterior to the coronal plane of the body by means of sling and swath. The supports were loosened three times a day to allow early shoulder motion up to 90 degrees of flexion and 0 degree of external rotation and full elbow motion. After 3 weeks, flexion and external rotation were gradually increased and with gradual stretching and improving subscapularis contracture we gained more external rotation. Internal rotation was begun at third weeks postoperatively.

## Evaluation

Patients' follow up was between 6 and 18 months with an average of 12 months. No patient was lost to follow up. The final functional results were rated at the time of the last follow up by the system proposed by Rowe and Zarins [10]. This is a point system (total possible point is 100) based on the assessment of pain, motion and function. Final motion was recorded at the end of follow-up period and we reported the loss of ROM by comparison to contra lateral shoulder. In all patients CT scan was performed before and after surgery. Postoperative CT scan was used to determine whether anatomic glenohumeral reduction had been achieved.

## Results

The overall Rowe and Zarin's score averaged 86 points. Four out of eight shoulders were graded as excellent, three as good and one as fair. The mean forward flexion and external rotation losses were 18 and 17.5 degrees respectively and the internal rotation loss was three vertebral body levels. Anterior active elevation averaged 140 degrees, external rotation 40 degrees and internal active rotation to the level of the 9^th ^thoracic body.

Table 2 shows the results of patients based on the Rowe and Zarin's score, range of motion and complications.

**Table 2:** Results of treatment based on Rowe score and range of motion

Case	Rowe score	External rotation loss(degree)	Internal rotation loss(vertebral body level)	Flexion loss(degree)	complications
1	100	0	0	0	None

2	85	40	2	30	Mild DJD

3	65	40	8	55	Subluxation

4	90	15	5	5	None

5	80	0	0	15	Subluxation due to subscapularis rupture

6	80	40	9	40	None

7	95	0	2	0	None

8	95	5	1	0	None

All patients were able to do their daily activities with mild or no pain. CT scan showed anterior subluxation in two patients. One of these cases had anterior glenoid bone defect involving one third of the joint surface and fair result in Rowe system. The other subluxated case had subscapularis insufficiency at belly press test and good result at the end of follow-up period (Rowe system). These two patients were able to perform daily activities with mild pain. Mild degenerative changes were present in one patient at final radiographs. Two patients had proximal head migration in their follow-up radiographs. One of them had surgically documented massive rotator cuff tear which was irreparable.

## Discussion

In reviewing the literature there are few studies about the results of operative treatment of chronic anterior shoulder dislocation. Most authors have recommended allograft reconstruction or arthroplasty in large head defects following chronic shoulder dislocation. Gavriilidis stated that shoulder arthroplasty resulted in good midterm results for 12 patients with severe head involvement with benefits for range of motion, pain and patient satisfaction [11]. The average duration of dislocation was 14 months in this report. In 13 patients with locked chronic posterior dislocation of shoulder and defect of between 25-50% of head, Diklic and coworkers reported good results with allograft reconstruction [12]. In our series Hill-Sach's defect was less than 40% and all were non-engaging. We suppose the reason is that the mean duration of dislocation in our cases (10 weeks) was less than that of the mentioned reports.

**Figure 2:**
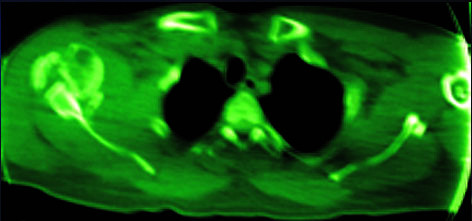
**CT scan of 17 week old anterior shoulder dislocation**.

**Figure 3:**
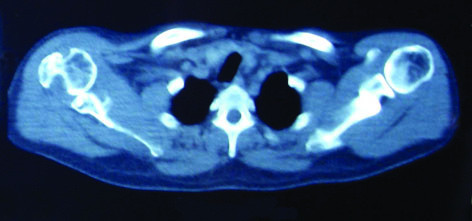
**Subluxation of shoulder duo to anterior glenoid bone defect 15 months after open reduction**.

One fair result in our study was in a case whose shoulder had Subluxation postoperatively. Anterior glenoid bone defect was the reason for subluxation in this patient which shows the necessity of bone grafting or coracoid transfer to the glenoid bone defects in such cases (Figure 2, 3). Perniceni and Augereau described reinforcement of the anterior shoulder complex in three patients after reduction of neglected anterior dislocation of the shoulder [13]. They used the Gosset technique [14] which places a rib graft between the coracoid and the glenoid rim.

Most reports have recommended shoulder joint transfixation to prevent redislocation following open reduction. Neviaser proposed transfixing the shoulder joint with a Swiss screw for three to four weeks [4]. Wilson and Mckeever recommended acromiohumeral crossed transfixing pins to prevent recurrence of the dislocation^5^. Rockwood and Green also suggested using smooth pins through the head into the glenoid for ten to fourteen days [6]. According to our study the results after capsulolabral complex repair appears to be more favorable than previously reported studies which have used metallic fixation methods. Postacchini et al reported good results in all four cases of operatively reduced chronic anterior and posterior dislocation [15]. Goga have reported three excellent, five good and two fair results in ten operatively reduced anterior shoulder dislocation [16]. Acromiohumeral k-wire fixation was used for 4 weeks in that group and the results were evaluated according to Rowe and Zarins system.

Supporting the arm at the side in a safe position was first stated by Rowe and Zarins in 1982 [10]. They recommended simply maintaining the arm at the side anterior to the coronal plane of the body for anterior dislocations and posterior to the coronal plane for posterior dislocations. In a report of seven operatively treated chronic shoulder dislocation with a mean duration of dislocation of 12 weeks, they had no postoperative dislocation using this simple method. Two shoulders were graded as excellent, three as good and two as fair with the mean Rowe score of 78 points.

**Figure 4:**
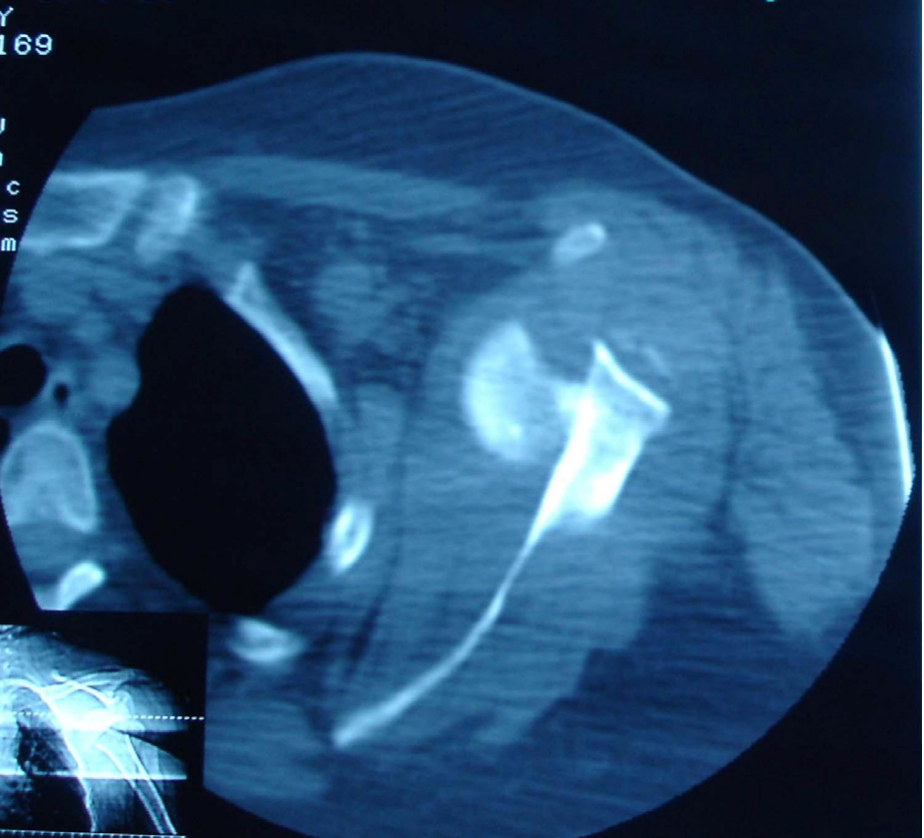
**CT scan of 5 week old anterior shoulder dislocation in a 65 year old woman And Forward flexion after 1 year**.

**Figure 5:**
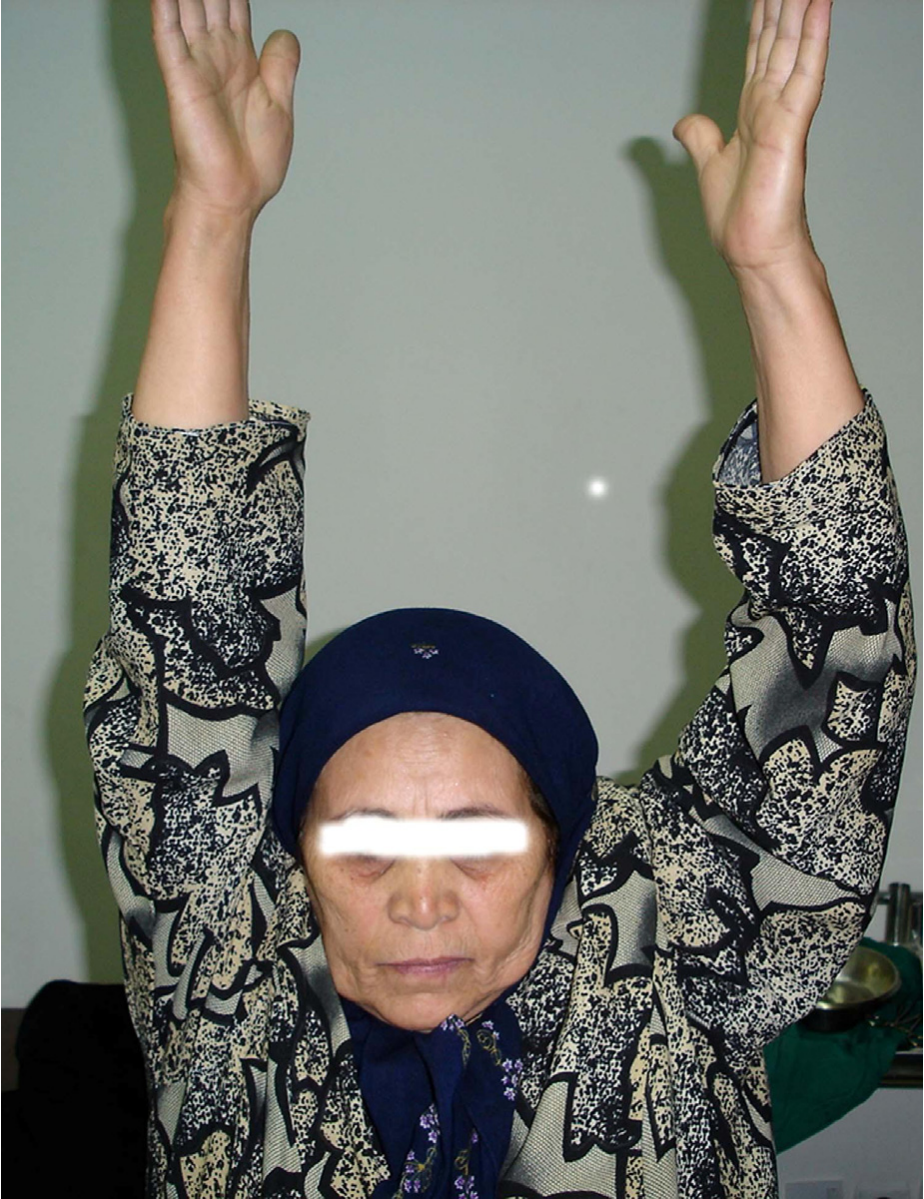
**CT scan of 5 week old anterior shoulder dislocation in a 65 year old woman And Forward flexion after 1 year**.

Capsulolabral complex repair allows early range of motion in a safe range without the fear of redislocation. We began up to 90 degrees of flexion and 0 degree of external rotation immediately in our patients. Although the average duration of dislocation have not pointed in Goga's study and it is difficult to compare his results with the present study, it seems that our patients as the patient in figures 4 &5 had much better range of motion at the end of follow up period and the average Rowe score in our patients was higher than Goga 's series. It should be mentioned that acromiohumeral fixation method had been used in Goga's study.

Our review of literature revealed just one report similar to our study. Mansat et al reported five patients with old anterior shoulder dislocation with average duration of 14 months [17]. All were treated with open reduction and capsulolabral insertion. At the end of follow up the average Rowe score was 75 points. The duration of dislocation in this group of patients was more than our study and this may be the reason for low Rowe score comparing with our series.

Mild degenerative joint changes were noted in only one patient. Although in the literature there is no report for the true incidence of osteoarthritis after operative reduction of old dislocations, it appears that early osteoarthritis rate is reasonable in our study and we think that the reason may be early motion and not using transfixing implants.

The present study had some important limitations. Although the present study is one of the largest reports in the literature it is confined to only eight patients. Another potential source of uncertainty in this study arises from the duration of follow up period. Longer follow up is needed for the detection of the true incidence of degenerative changes following open reduction of old shoulder dislocations.

In conclusion the authors of this article recommend concomitant open reduction and capsulolabral complex repair, when possible, in the treatment of old anterior shoulder dislocations.

## Competing interests

The authors declare that they have no competing interests

## Consent

Consent was obtained from the patient for publication of this report and accompanying image.

## Authors' contributions

Author AR and AN performed surgeries - AR performed follow-ups - AN performed design of the study - AN performed statistical analysis of the study - AR participated in the sequence alignment and drafted the manuscript. Both authors have read and approved the final manuscript.

## References

[B1] Bennett GE Old dislocations of the shoulder J Bone Joint Surg 1936 18 594 606

[B2] Mirick MJ Clinton JE Ruiz E External rotation method of shoulder dislocation reduction J Am Coll Emerg Physisicians 1979 8 528 31 10.1016/S0361-1124(79)80302-0 513410

[B3] Engel T Lill H Korner J Josten C Bilatera fracture dislocation of shoulder caused by an epileptic seizure -diagnostic, treatment and result Unfallchirurg 1999 102 11 897 901 10.1007/s001130050499 10551938

[B4] Neviaser JS Treatment of old unreduced dislocations of the shoulder Surg Clinic North America 1963 43 1671 1678 10.1016/s0039-6109(16)37160-2 14090219

[B5] Wilson JC McKeever FN Traumatic posterior (retroglenoid) dislocation of the humerus J Bone Joint Surg 1949 31 A 160 72 18122881

[B6] Rockwood C Green DP Fracture 1975 Philadelphia:J.B Lippincott 710 8

[B7] O'Hara BP Urban JP Influence of cyclic loading on the nutrition of articular cartilage A MaroudasAnn Rheum Dis 1990 49 536 539 10.1136/ard.49.7.536 PMC1004145 2383080

[B8] Rubak Jens M Poussa Mikko Ritsilá Veijo Effects of Joint Motion on the Repair of Articular Cartilage with Free Periosteal Grafts 1982 53 2 187 191 6753459 10.3109/17453678208992199

[B9] Bankart ASB Recurrent or habitual dislocation of the shoulder joint Br Med J 1923 1132 1133 10.1136/bmj.2.3285.1132 PMC2317614 20771383

[B10] Rowe CR Zarins B Chronic unreduced dislocation of the shoulder J Bone Joint Surg 1982 64 4 494 505 7068692

[B11] Diklic ID Ganic ZD Blagojevic ZD Nho SJ Romeo AA Treatment of locked chronic posterior dislocation of the shoulder by reconstruction of the defect in the humeral head with an allograft J Bone Joint Surg Br 2010 92 1 71 6 10.1302/0301-620X.92B1.22142 20044682

[B12] Gavriilidis I Magosch P Lichtenberg S Habermeyer P Kircher J Chronic locked posterior shoulder dislocation with severe head involvement Int Orthop 2010 34 1 79 84 10.1007/s00264-009-0762-9 19300999 PMC2899272

[B13] Perniceni B Augereau A Treatment of old unreduced anterior dislocations of the shoulder by open reduction and reinforced rib graft: discussion of 3 cases Ann Chir 1983 36 235 9 7092118

[B14] Gosset J Une technique de greffe coraco-glenordienne dans le traitement des luxations recidivantes de l'epoule Mem Acad Chir 1960 86 445 713851387

[B15] Postacchini F Facchini M The treatment of unreduced dislocation of the shoulder. A review of 12 cases Ital J Orthop Traumatol 1987 13 1 15 26 3692794

[B16] Goga IE Chronic shoulder dislocation J shoulder Elbow Surg 2003 12 5 446_50 10.1016/S1058-2746(03)00088-0 14564265

[B17] Mansat P Guity MR Mansat M Bellumore Y Rongieres M Bonnevialle P Chronic anterior shoulder dislocation treated by open reduction sparing the humeral head Rev Chir Orthop Reparatrice Appar Mot 2003 89 1 19 26 12610432

